# High-Pressure Hand Injection Injury Case Report

**DOI:** 10.21980/J8NM0P

**Published:** 2020-04-15

**Authors:** Mary Rometti, Patricia Mangel

**Affiliations:** * Rutgers Robert Wood Johnson Medical School, Department of Emergency Medicine, New Brunswick, NJ

## Abstract

High-pressure injection injuries present to trauma centers a few times each year, typically involving the patient's non-dominant hand.^1^–^6^ Symptoms may include progressive pain, numbness, swelling of the affected area, and decreased range of motion.^2^,^6^–^8^ These injuries are important to recognize because the initial injury can involve damage to ligaments, tendons, and neurovascular structures.^1^–^5^ Pressure increases, from the injected material and subsequent tissue swelling, can result in vascular injury and compartment syndrome.^1^,^2^,^5^ Antibiotics and prompt surgical evaluation are vital because high-pressure injection injuries are considered a surgical emergency.^1^,^2^,^7^–^9^

**Topics:**

High-pressure injection injuries, hand injuries, orthopedics

[Fig f1-jetem-5-2-v4]

**Figure f1-jetem-5-2-v4:**
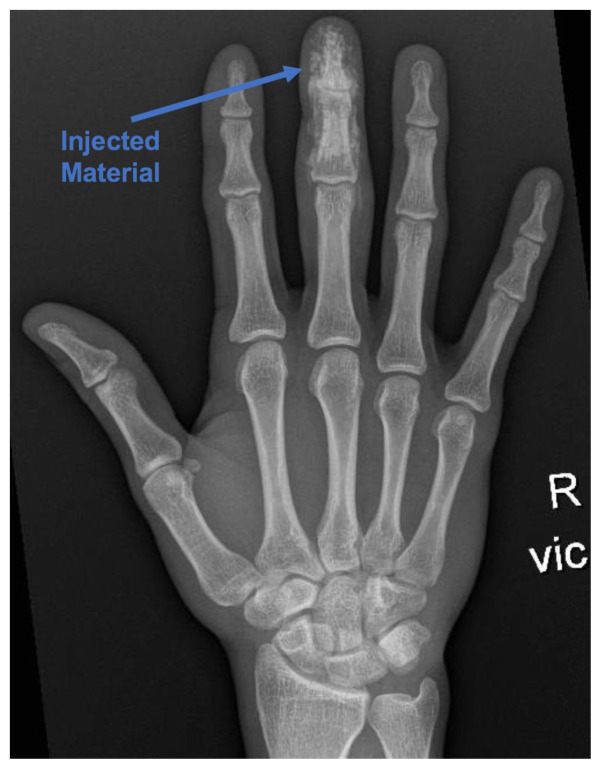

[Fig f2-jetem-5-2-v4]

**Figure f2-jetem-5-2-v4:**
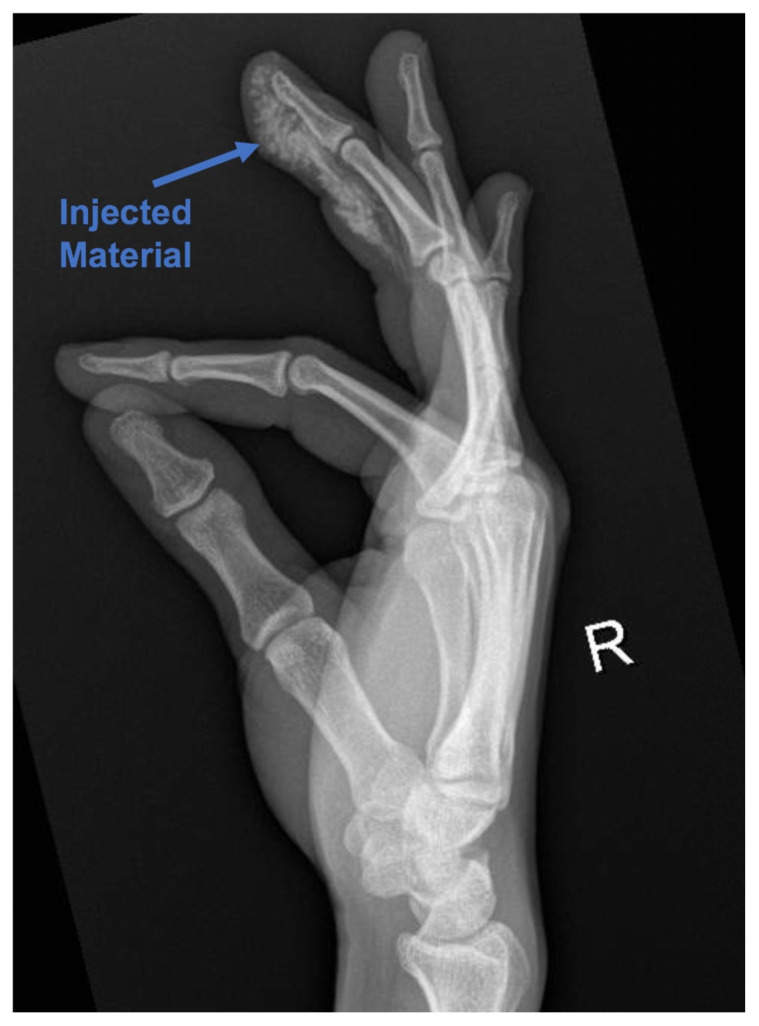

## Introduction

High-pressure injection injuries cause approximately 1 in 600 traumatic hand injuries.^1^–^5^ A trauma center receives approximately one to four cases of high-pressure injection injuries per year.^1^–^3^,^5^,^6^ These injuries most commonly occur in the patient’s non-dominant second digit.^1^,^2^,^4^,^5^,^6^ Often, high-pressure injection injuries are work related.^1^,^2^,^5^,^8^ Injection injuries may be misleading because the injury may initially appear minor.^1^–^6^,^10^ Emergency physicians must be able to quickly identify and initiate prompt treatment.

## Presenting concerns and clinical findings

A 23-year-old male presented to the emergency department (ED) one hour after injuring his right middle finger at work, while using a high-pressure paint sprayer containing exterior latex paint. He instantly felt pain, numbness, and swelling in his affected digit and initially was able to express paint from the finger. On exam, the punctate injection site was visible on the right third digit finger pad with paint covering a majority of the distal right upper extremity. The right third digit was held in partial flexion. Numbness extended distally from the right distal interphalangeal (DIP) joint. Motor exam revealed limited flexion and extension of the DIP joint. The remainder of the right digits were without obvious injury and exhibited full strength and sensation on exam.

## Significant findings

X-rays of his right hand revealed extensive infiltrates of the right distal and middle phalange without fractures or dislocations.

## Patient course

Orthopedic hand surgery was consulted in the ED. Cefazolin and pain medication were given. Tetanus was not needed because the patient was up-to-date. The patient was admitted for an initial irrigation and debridement (I&D) that day. Two days later, he had his second I&D to expose healthy tissue, remove additional paint, and irrigate the area with crystalloid. Following this second procedure, he was discharged and instructed to continue antibiotics for one week. His right third digit remained numb but with brisk cap refill. Sensation to light touch was intact for his remaining digits. A volar resting splint was placed. Follow-up was scheduled with orthopedic surgery four days later.

## Discussion

During a high-pressure injection injury, the initial force drives high-pressure materials such as air, paint, or grease along a path of least resistance which often extends around the neurovascular structures, ligaments, and tendons.^1^–^5^ The resulting increase in pressure may lead to tissue swelling, vascular injury, and compartment syndrome.^1^,^2^,^5^ Following the initial injury, an inflammatory response is mounted to the foreign material which can create additional tissue swelling and an increase in pressure.^1^–^3^,^5^,^6^

Treatment begins with pain medication, starting prophylactic antibiotics, and a tetanus booster if patient is out of date.^1^,^2^,^4^–^8^ Often, third generation cephalosporins are used to cover gram-negative and gram-positive bacteria.^1^–^3^,^5^,^6^ Other literature suggests using first generation cephalosporins or gentamycin.^3^,^8^ While an infection can occur from the initial injection injury, a secondary infection incited by tissue necrosis and ischemia is more likely.^5^ Obtaining radiographs of the affected area provides insight into the extent of damage if the injected material is radiopaque or radiolucent air is visible.^4^,^5^,^8^–^10^ Initial ED treatment should be directed at elevating the injury site, copiously irrigating the wound, and emergently consulting surgery. The wound should not be closed because the injury is likely to swell.^2^ Prompt surgical intervention is needed to irrigate and debride the wound, and to decrease compartment pressures.^1^,^2^,^7^ Often a second debridement is needed after 48–72 hours to remove additional unhealthy tissue or injected material.^2^,^3^ If surgery is not available, the patient should be transferred to an appropriate trauma center.^2^ High-pressure injection injuries are a surgical emergency.^9^

Possible complications include decreased function of the injured limb, subsequent infections, paresthesias, contractures, chronic pain, or necessary amputation of the limb.^2^,^4^,^6^ Multiple factors affect the clinical course and the need for amputation, such as the injection site and injection material. When the injection injury involves the fingers, there is almost a three-fold increase in the likelihood of an amputation, as compared to the thumb or palm.^10^ Furthermore, the type of injection material impacts outcome. One review found that organic solvents, such as paint or gasoline, require an amputation over 40% of the time.^10^ Surgery is often not required when water or air is the injected material because this is associated with a better prognosis.^2^,^10^

This case highlights the importance of recognition and quick intervention in high-pressure injection injuries. A limitation of this case is that the patient was not followed for a longer period of time to see what further complications may have resulted from his injury. While from the surface, injection injuries may appear minor, the resulting damage within the tissue can be quite extensive.

## Supplementary Information








