# Supplementary figures and images for: High-Pressure Hand Injection Injury Case Report
Source: J Educ Teach Emerg Med. 2020 Apr 15;5(2):V4–6. doi: 10.21980/J8NM0P (PMC10332568; doi:10.21980/J8NM0P)

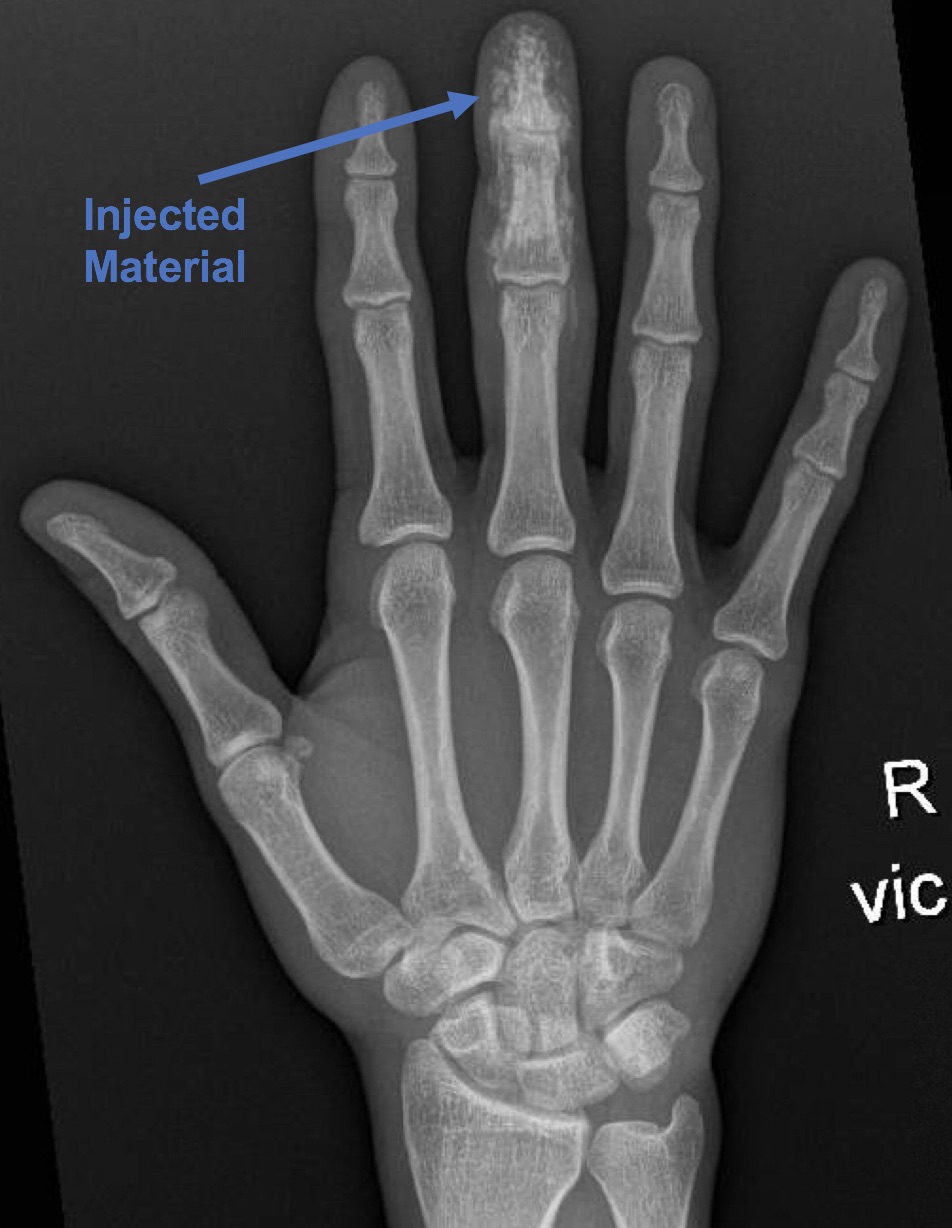

Supplement: Supplementary file 1 [file jetem-5-2-v4-supp1.jpg]

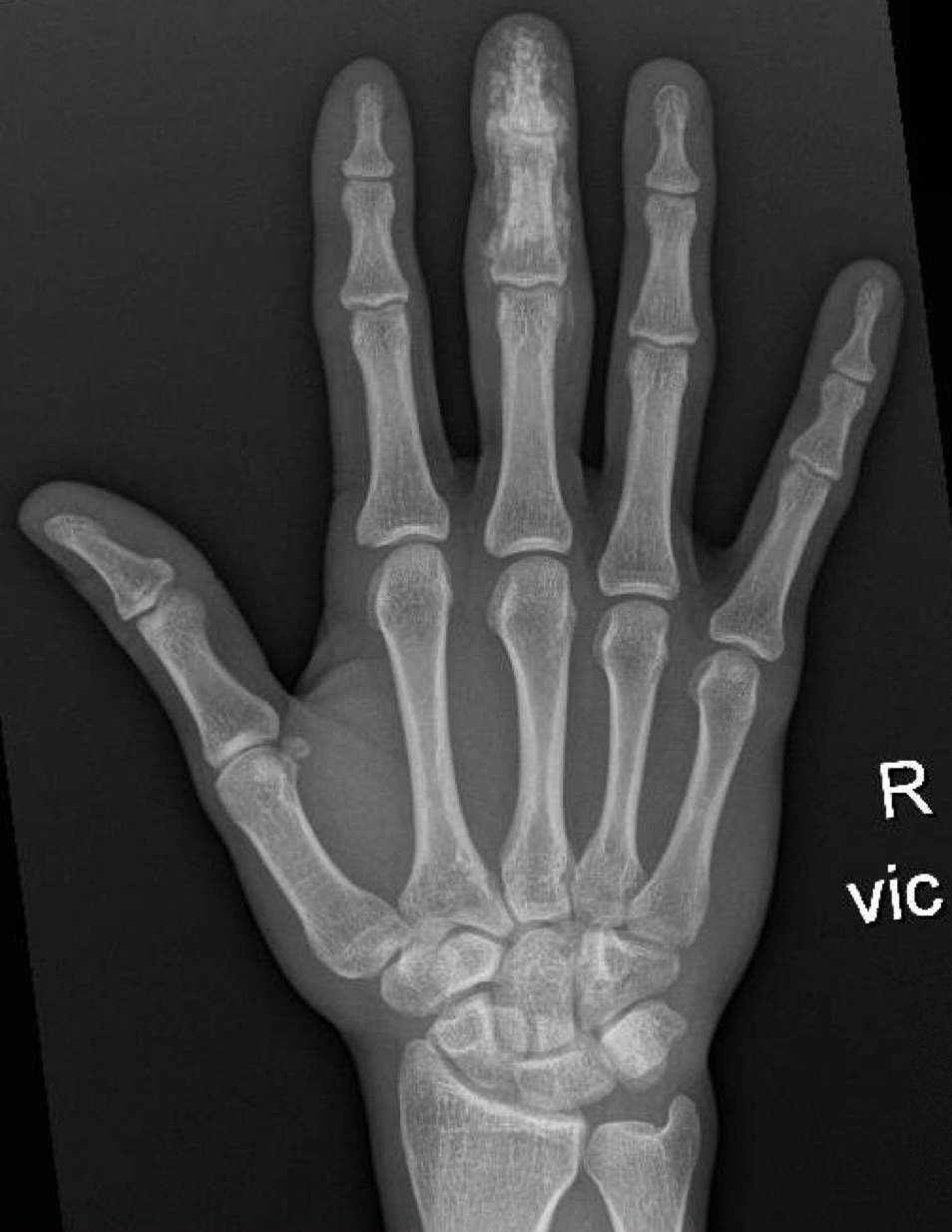

Supplement: Supplementary file 2 [file jetem-5-2-v4-supp2.jpg]

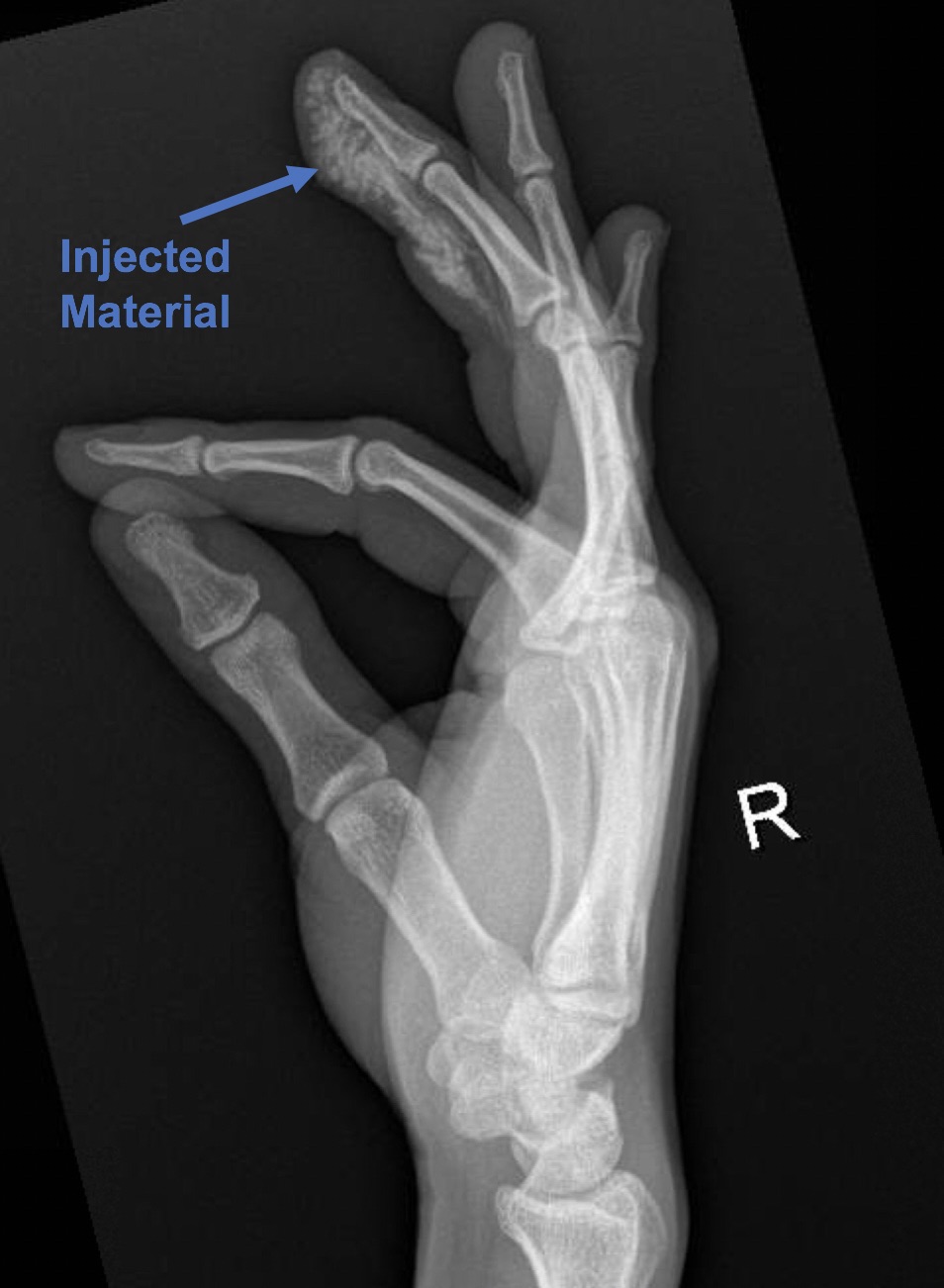

Supplement: Supplementary file 3 [file jetem-5-2-v4-supp3.jpg]

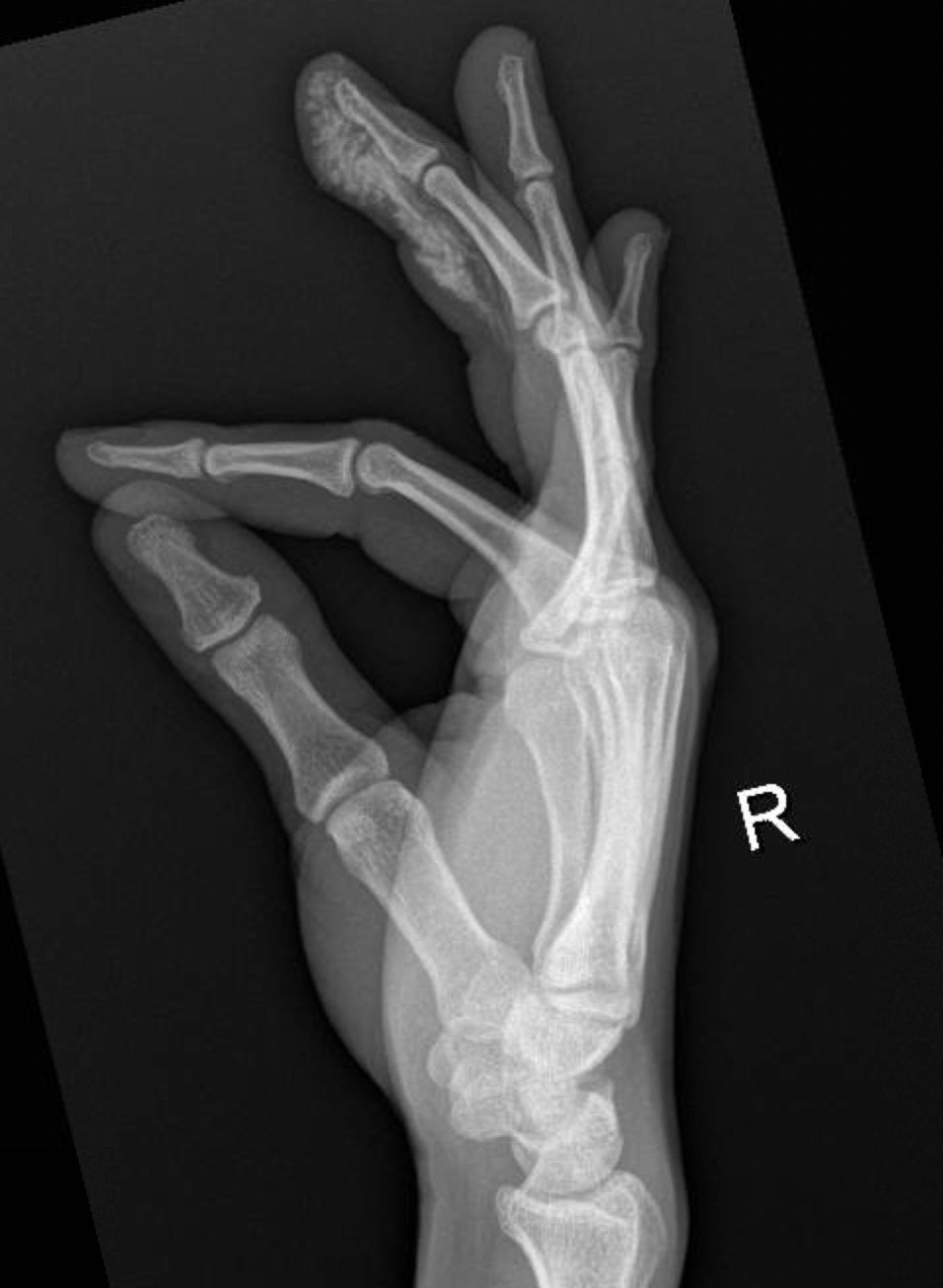

Supplement: Supplementary file 4 [file jetem-5-2-v4-supp4.jpg]
